# Locked–in Syndrome in a Nigerian male with Multiple Sclerosis: a case report and literature review

**Published:** 2008-10-30

**Authors:** Imananagha Kobina Keme-Ebi, Asindi Asindi Asindi

**Affiliations:** 1 Department of Medicine, Neurologic Unit, Niger Delta University Teaching Hospital, Okolobiri, Nigeria;; 2 Department of Paediatrics, Neurologic Unit, University of Calabar Teaching Hospital, Calabar, Nigeria;

**Keywords:** Locked-in Syndrome, Nigeria

## Abstract

**Background:**

Locked-in syndrome is an extremely difficult neurologic condition to recognize, especially by the non-specialists. A case of locked-in syndrome in a 41–year old Niger Deltan Nigerian with relapsing remitting form of multiple sclerosis (MS) is presented, including a detailed literature review.

**Patient and case report:**

The patient was in a state of spastic quadriplegia, motionless and aphasic (mute), with the preservation of consciousness and the ability to open and blink the eyes and move them vertically. Two episodes of the disease, varying in duration, have been described. The diagnosis of MS was made from the history and the typical clinical presentation: history of relapsing and remitting signs and clinical evidence of multi-focal involvement of the central nervous system.

**Conclusion:**

Patient died at the age of 45 years, from pulmonary complications. This article may enhance easy recognition and management of the syndrome by all clinicians.

## Background

Locked-in syndrome (LIS) is a rare and difficult condition to recognize by clinicians, especially the non-neurologists.[1–33] This syndrome poses serious challenges to the clinicians both in terms of the diagnosis and management, for it may be confused with stroke and other causes of unconsciousness. To the non-specialist doctor the patient appears unconscious; such a belief may lead the clinician to a wrong diagnosis and inappropriate management of the patient, especially if the doctor fails to examine vertical ocular movements. Another danger is that the attending doctors and nurses may discuss the “unconscious” patient’s condition at the bedside, to the hearing of the patient, which may enormously stress the patient and cause deterioration of the condition.

The syndrome is characterized by quadriplegia and anarthria in a patient with preserved consciousness: [2–13]. The patient is motionless and mute, but retains the ability to open and blink the eyes and move them vertically. The diagnosis in most cases, is made after a family member or hospital nursing staff calls the attention of the doctor to the fact that the patient, who appears comatose is awake and aware of his immediate surroundings or if MRI shows a ventral pontine lesion, coupled with a normal EEG in an otherwise unresponsive patient. It is known that cerebral infarction or haemorrhage, brainstem trauma, demyelinating disease, tumours and encephalitis [5–7;14] can cause LIS, especially when these lesions affect the ventral portion of the lower pons and medulla, although demyelinating diseases are rare in the African continent. The prevalence of MS is less than 1 per 100,000 in the equatorial area (e.g. Nigeria) and blacks are at lower risk than whites in all latitudes. There are no reports in the literature of LIS in an African with multiple sclerosis. We saw such a patient in our practice at the University of Calabar Teaching Hospital, Calabar, Nigeria, whom we followed-up until his death. We hereby report this interesting case, to enhance easy recognition of the syndrome by all clinicians, especially those practicing in developing countries.

## Patient and case report

### 1. The First Episode

A 41 – year old Nigerian male university lecturer was previously well until the day his wife first noticed some degree of unsteadiness in his gait, while walking. This did not bother him until about 2 months later when he complained of weakness in all four limbs. Days later, while waiting to address a conference, the weakness suddenly became so profound that someone else had to take–over the reading of his prepared conference paper. At the end of the conference, he was driven home by his wife, as he was too weak to do so himself. Few hours later, when he became completely paralyzed in all 4 limbs and could no longer talk, he was taken to the University of Calabar Teaching Hospital, Calabar, Nigeria, where he was admitted the same day. His past medical history contained nothing that could point to the cause of his illness: no history of hypertension, diabetes mellitus, stroke, head injury, psychiatric illness or other chronic illnesses or recent neuro-infections. He was married with four children.

On physical examination: patient was lying motionless, with opened eyes, which blinked occasionally. He could not speak, but tried to communicate with the eyes, which could move in the vertical plane only. Patient reacted to what people said and other happenings in his immediate environment, by moving the eyes or blinking them; meaning that the motionless and mute patient was indeed conscious. The chest: no respiratory distress, clinically clear; BP: 130/80 mm Hg; abdomen: no abnormalities. Neurological status: decerebrate posturing; signs of corticospinal tract dysfunction– quadriplegia, spasticity and exaggerated deep tendon reflexes in all 4 limbs; and bilateral cranial nerves palsies (3rd, 6th and 7 ^th^ ). Based on the above clinical findings, a diagnosis of locked – in syndrome (cause?) was made. On admission: full blood count, red cell sedimentation rate, blood sugar, urea, electrolytes, creatinine and liver function were all normal. VDRL was negative. On the 2nd day, before other planned investigations (e.g CSF and x–ray examinations etc) could be conducted, patient was taken out of hospital by his family members, against medical advice. At home, because of the “mutism”, suspected to be due to depressive psychoses, a patient’s relation, a pharmacist, on her own initiative, introduced an anti –depressant drug to the therapy, with no positive effect. Next they took him to a pastor of a church, for divine healing.

The patient was in the motionless– mute state for 5 months. From the 6th– 8th months, according to the wife, without further medical care, apart from occasional physiotherapy, functions gradually returned: initially movements in the upper limbs resumed, followed by ability to sit up and the use of his lower limbs. Later the speech gradually improved. About a year after the onset of illness, patient, not discouraged by residual neurologic deficits (namely, spastic gait and speech disorder), resumed duty at his workplace.

A repeat neurological evaluation 7 months after he resumed duty revealed intact cognitive functions. However, he complained of unsteadiness in walking, hearing difficulty, with buzzing sound in both ears like the sound of an approaching aeroplane. He could not recall most of the events of the past months of his illness. On examination: mild quadriparesis with dysarthria; audiometric test: bilateral sensorineural deafness (worst on the left); fundoscopy: bitemporal pallor; computerized tomography (CT) of the brain showed slightly dilated lateral ventricles, without suggesting its definitive cause in this particular case: normal EEG.

The buzzing sounds in the ears cleared completely by the 21st month of illness; but hearing difficulty and unsteady gait persisted. Patient had to wear hearing aids.

### 2. Second Episode

In the 32nd month of illness, patient suffered yet another attack: unsteady gait, severe weakness of the four limbs and profound speech disturbance. Generally, the presentation reminded the family members of the 1st attack. Consequently the family once again called in the Pastor of his Church, for spiritual healing. Three weeks later, when patient’s condition further deteriorated, he was brought to the hospital. Neurological evaluation revealed: dementia; a motionless patient, quadriplegic, unable to talk, sit-up or walk; painful flexor/extensor spasms; cranial nerves palsies (3rd, 6th and 7th). The relapsing nature of the disease, with symptoms/signs indicative of multi–focal central nervous system involvement, together with the failure of CT scan to identify lesion suggestive of pontine haemorrhage or infarction, tumour or brainstem trauma, raised the suspicion, that ventral pontine demyelination (multiple sclerosis) may be the most likely cause of our patient’s Locked – in Syndrome, and that the attacks (episodes 1 and II) were exacerbation of the multiple sclerosis. Prompted by this realization, the management of the case was reviewed, and included (i) corticosteroid (prednisolone) therapy and (ii) Baclofen, for the treatment of spasticity and the painful flexor/extensor spasms. In the next two months, patient’s condition gradually improved, the spasticity became less incapacitating with time. The speech too improved during the 2 – month stay in hospital. But in one occasion, patient’s condition deteriorated drastically: for the first time, during the course of illness, he lost consciousness, for about 6 hours. Investigations revealed; (i) normal chest x-ray (excluded pneumonia) and (ii) asexual *P. falciparum* parasitaemia (confirmed severe malaria as the trigger for the deterioration of the patient’s condition). Patient responded dramatically to a course of anti-malaria drug therapy (with fansidar): regained full consciousness within 12 hours. Satisfied with the level of improvement of his mental state, motor function and speech, patient was discharged home by his doctors, at the end of a 2-month stay in the hospital. By the 4th month, the patient had recovered most functions and was well again.

### 3. Death of Patient

8 months after discharge from hospital, patient’s condition once again deteriorated: at first he was febrile; and later became quadriplegic and comatous. He was rushed to a nearby private hospital, where chest x-ray examination revealed pneumonia. Before appropriate antibiotic therapy could be initiated the patient died. The family did not allow autopsy.

## Discussion

### Case Definition

Emile Zola, [15] a non-medical writer, was the first in 1868, to describe a woman with this syndrome in a book “Therese Raquin”. However, it was more than hundred years later that Plum and Posner [16] introduced the term “Locked-in Syndrome”.

LIS was first re-defined in 1966 as quadriplegia, lower cranial nerve paralysis and mutism, with preservation of consciousness, vertical gaze and upper eyelid movements [16]. So that mutism would not be mistaken for unwillingness to speak,[17] in 1986, LIS was again re-defined as a syndrome characterized by quadriplegia and anarthria, with preservation of consciousness.[18]

The locked-in patient is usually conscious, while lying mute and motionless. He retains blinking and voluntary vertical eye movements, which facilitate non-verbal communication [1–14, 19]. That means, the patient is aware of his surroundings, but is physically locked-in.[12, 13] Some cases may exhibit decerebrate posturing.[6]

One could logically refer to this state as akinetic mutism since the patient is akinetic (motionless) and mute, but this condition originally coined by Cairns [12], is somewhat different in that the lesion of akinetic mutism lies in the cerebral hemispheres hence the patient is unaware of his surrounding but shows sleepwake-cycle and may be able to withdraw from a painful stimulus.

### Pathologic Basis of LIS

The pathophysiology of LIS involves a lesion affecting the ventral portion of the lower pons or medulla with the sparing of the upper pontine tegmentum.[6;14] The diencephalons and the ascending reticular formation responsible for consciousness lie above the mid pons hence consciousness is preserved in these patients. Thrombosis of the upper segment of the basilar artery resulting in infarction, haemorrghage, trauma (brainstem contusion or vertebro-basilar axis dissection), viral brainstem encephalitis, central pontine myelinolysis, demyelinating disease (acute or chronic relapsing multiple sclerosis), and primary and secondary tumours, all affecting the ventral pons or medulla, are the usual causes of locked-in syndrome [2, 6–9, 13, 14]. There are reports also, that severe Guillian Barre Syndrome [20] and snakebite [21;22] may cause LIS.

A lesion at the mid pontine level, while sparing the somatosensory pathways, may interrupt [2–5, 17, 18, 23, 24] the corticobulbar and corticospinal tracts to the lower cranial nerves and the limbs, resulting in the paralysis of all four limbs, face and pharynx and larynx. The bilateral facio-glosso-pharyngo-laryngeal paralysis [25] causes anarthria, dysphagia and limits the use of the muscles of facial expression in communication [2]. These factors may explain why the locked-in patient is motionless, quadriplegic and mute, but conscious, as it was the case with our patient.

Vertical eye movements are controlled from a centre in the superior collicular region of the midbrain and are thus preserved together with the pupillary light reflex [13]. A mid pontine lesion is likely to affect the abducens nucleus and cause the loss of lateral gaze, which is a feature of the disease as manifested in this case. The preserved partial eye control can therefore be utilized to affirm awareness in the patient by ordering him to open and close the eyes.

The ataxia associated with this condition and experienced by our patient most probably arises from the involvement of the pontine nuclei and the fibers which connect the pons with the cerebellar hemispheres. The lower part of the pons contains a number of neurons and fibres connecting the pontocerebellar and corticopontine tracts. All voluntary movements originate from the cerebral cortex with simultaneous transmission to the cerebellar cortex via the nuclei pontis. The activities of the cerebellar cortex initiated in this way are immediately relayed back to the cerebral cortex via the superior cerebellar peduncles. Any lesion affecting this circuit is bound to present with ataxia.

This patient also experienced buzzing sound in both ears, which took more than two years to clear, and sustained a permanent hearing defect in both ears. These auditory disturbances may suggest pontine auditory hallucinosis (i.e. complex auditory illusions with some qualities of hallucination, which may accompany pontine lesion). Auditory hallucinosis consists of alternating musical tones, like an organ or a jumble of sound (like a symphony Orchestra tuning up) or siren- like or buzzing sound, like a swarm of bees.

These auditory sense disturbances are more complex than neurosensory tinnitus but less formed than temporal lobe hallucinations. They are usually associated with impairment of hearing in one or both ears and other neurologic signs related to the pontine lesion (as in our patient). Brainstem evoked potentials may reveal intact cochlear, auditory nerve and cochlear nuclear responses, although our patient showed a bilateral sensorineural deafness.

The mild dilatation of the lateral ventricles in this case may indicate some degree of obstruction of the ventricular system; but we cannot use this to explain LIS in our patient as the computerized tomography did not suggest the actual cause of this dilatation and there was no history to suggest tumour or support a vascular aetiology (cerebral haemorrhage or infarction) of LIS in our patient.

### Diagnosis

#### Locked –In Syndrome

The diagnosis of LIS [1, 2, 5, 33], in most cases, is made when family members or hospital nursing staff call the attention of doctors to the fact, that the patient, who appears comatous, is awake and aware of his immediate surroundings or if MRI shows a ventral pontine lesion, coupled with a normal EEG, in an otherwise unresponsive patient. In such cases, it is mandatory for the doctor to assess voluntary eye opening and blinking abilities and vertical eye movements [6]. These brainstem- mediated movements are preserved in LIS. CT scanning, MRI and auditory evoked response are some of the sensitive tests that can be used to investigate the brainstem of LIS patient. Unfortunately, we did not have these facilities in our practice. A conventional EEG with stimulation can distinguish between LIS (normal EEG, as it was with our patient) and coma (abnormal EEG).

#### Multiple Sclerosis (MS)

Initially the MS patient presents with an unsteady gait, spastic paraparesis, retrobulbar neuritis, diplopia and sphincter dysfunction [6, 14].All or most symptoms may disappear after a few days/weeks/months. Our patient presented with most of these initial symptoms. Infections, trauma or pregnancy can trigger exacerbation of this disease. Diagnosis of MS is based on the total clinical picture that indicates the involvement of different parts of the central nervous system (CNS) at different times. [6, 14]

The multiple CNS lesions in MS are detectable by electrocerebral responses evoked by: (i) monocular visual stimulation with checkerboard patterns (visual evoked potentials, VEP); monaural stimulation with repetitive clicks (brainstem auditory evoked potentials, BAEP); and electrical stimulation of a peripheral nerve (somatosensory evoked potentials, SSEP) [6;19]. MRI too has become nearly indispensable in confirming the diagnosis of MS [6]. These facilities were not available for the management of our patient.

The Cerebro-Spinal Fluid (CSF) is commonly abnormal [6]: mild lymphocytosis or slightly increased protein concentration, especially soon after an acute relapse. Spinal tap was not done, because the relations did not create the opportunity for us to do this relatively simple procedure. CSF protein electrophoresis could have shown the presence of discrete bands in the 1gG region (oligoclonal bands), as this is the case in 90% of MS Patients [6]. The diagnosis of MS in our patient was made from the history and the typical clinical presentation. The illness took about two months to evolve and partial recovery from the first episode took several months. The history of relapsing and remitting signs of corticospinal tract dysfunction and the clinical evidence of disseminated lesions in the CNS led us to the diagnosis of a demyelinating disease (due to a slow virus), probably MS as the probable cause of LIS in our patient.

The prevalence [7] of MS is less than 1 per 100,000 in the equatorial (e.g. Nigeria).There is increasing risk of development of MS with increasing latitude. Blacks are at lower risk than whites at all latitudes. From the history, it was known that our patient once lived in Britain for several years. Whether this had anything to do with the development of MS in our patient was difficult to establish.

### Classification

#### Locked–in Syndrome

LIS [5] has been classified into three (3) categories [26]:

a. Classic–quadriplegia and anarthria with preserved consciousness and vertical eye movement; (b) Incomplete – the same as the classic form, but with remnants of voluntary movements other than vertical eye movement; (c) Total–total immobility, including absence of eye movements, and inability to communicate, but fully consciousness. These 3 categories are further subdivided into transient and chronic forms [26].Clinically our patient had classic LIS (episodes I and II). Although there is no specific classification system for vocal, cognitive, emotional, dysphagia or behavioural recoveries; there exists one for recovery of motor function in LIS [5]: (a) No recovery– no return of motor function, total dependence for all activities of daily living; (b) Minimal recovery– minimal motor return, total dependence for all activities of daily living; (c) moderate recovery – moderate motor return, independence in some, but not all activities of daily living; (d) full recovery – independence in all activities of daily living, but some minimal neurological deficits ; (e) No neurological deficit – no reported residual deficits. At the end of episode I, our patient experienced almost a full recovery; and episode II, moderate recovery.

#### Multiple Sclerosis

The fact that MS is undoubtedly a rare disease in the African continent (and as the probable cause of LIS in our patient) makes this case very interesting. Clinically, MS can take any of the following forms: [6, 7, 20] (a) Mixed (or generalized) type. i.e. cerebrospinal type (50% of the cases);(b) Spinal form (30–40%); (c) predominantly pontobulbar–cerebellar or cerebellar form (5%) and (d) amaurotic form (5%). The ponto–cerebellar form has a rapid course, leading to severe invalidism in 4 years. We believe the cause of LIS in our patient was predominantly pontobulbar–cerebellar MS, [6, 19] which might have affected the ventral pons. There is yet another classification of MS. 3 clinical forms of MS: (a) Relapsing-Remitting Form- clinical progression does not occur between the attacks. There is an interval of months or years of no clinical activity after initial episode before new symptoms develop or original ones reoccur; (b) Secondary Progressive Form– characterized by a gradual progressive course after initial relapsing–remitting pattern. The disease relapses; usually with incomplete remissions. Steady deterioration leads to increasing disability with weakness, spasticity, ataxia, impaired vision and urinary incontinence; (c) Primary Progressive Form – gradual progression of disability from the clinical onset. Most likely our patient suffered from relapsing – remitting form of MS.

### Management

#### LIS Management

Acute Management [2] is similar to the care of the unconscious patient or to that for patients with other brainstem aetiology: airway maintenance, adequate oxygenation, adequate food and fluid intake via intravenous infusions and nasogastric tubes, urine and bowel output drainage and clearing up; frequent turning of patient to prevent formation of bedsores, physiotherapy to prevent stiffness of joints and deep vein thrombosis (chest physiotherapy must include deep breathing exercises) suctioning, postural drainage, risk reduction, management of treatable causes, treatment of corneal ulceration due to impaired eye closure (lateral tarsorrhaphy or botulinum therapy), treatment of pathological crying, with selective serotonin reuptake inhibitors.

Medical and Nursing staff must not make casual remarks at the patient’s bedside –it may cause the patient mental anguish [27]. Since the patient can not communicate discomfort or wants, his/her needs must be anticipated and met by diligent family members on hospital staff. Patient’s needs include companionship and variety of daily experiences that a person would crave. During rehabilitation, monitor for recovery of thumb, finger, head and neck movements; swallowing independently, and improved respiratory function.[2] Aggressively treat infections, respiratory difficulties and pains. Patient – computer interfaces [24] (e.g. infrared eye movement sensors and computer voice prosthetics) and augmentative communication devices are being developed to assist the LIS patient communicate better [28]. Patients with LIS are increasingly being managed in specialized centres that have experienced manpower and equipment.

#### Management of MS

- Corticosteriod therapy: this hastens recovery from acute relapse. Long term usuage is not advisable, as it does not prevent relapses. I.V methy1-prednisolone, 500–1000 mg daily for 3 – 7 days. [6, 29]
- Prophylactive drugs [6, 19, 29, 30]: These drugs delay onset of significant disability in patients with relapsing disease: Beta – interferon – 1b (Betaseron)- 8 Million units, subcutaneously, on alternate days (flulike side effects); Beta– interferon– 1a (Avonex)- 6 million units/weekly, intramuscular injections; Glatiramer acetate (copaxone)-20mg /daily, subcutaneously. Immunosuppressive therapy with– cyclophosphamide, azathioprine, methotrexate, cladribine, or mitoxantrone may help arrest the the course of secondary progressive MS [14, 29].
- Management of neurological deficits [14, 19; 31]: (a) fatigue –Amantadine (symmetrel)–160 mg twice daily; or pemoline (cylert)–18.75 mg twice daily. The drugs reduce fatigue; (b) motor deficit – little can be done to restore muscle strength; spasticity– baclofen (lioresal)- 60 mg /daily, or Tizanidine (Zanaflex)-8 mg, 4 times daily, or oral diazepam, physiotherapy; (c) urologic problems: manage the hyperreflexic bladder, with small capacity (urinary frequency and incontinence)– oxybutinin (Ditropan), Pro-Banthine. Flaccid bladder – self – catheterization.

### Prognosis

LIS mortality is about 60%, greatest in the first 4 months of illness, higher (70%) in patients with vascular lesion than non-vascular causes (40%) [5].Ten year survivor rates as high as 80% have been reported [2]. Even limited physical recovery can improve quality of life and enable the patient to return to live with their families. Early multidisciplinary [3, 5, 17–24] rehabilitation and effective nursing care reduces mortality from acute LIS. [3] Young patients survive better. Survivors may recover partially or completely over a period of weeks or months. The following are some possible causes of death of LIS patients;

- Pulmonary complications are the leading causes of death e.g. aspiration of saliva, due to dysphagia and impaired cough reflex leads to atelectasis and pneumonia. Immobility predisposes to pulmonary embolus [32].
- Death from primary destructive lesions (the lesion that caused the syndrome): cerebral infarction, haemorrhage, demyelination, trauma, encephalitis, all involving the ventral pons and sparing the tegmentum. Our patient died from pulmonary complication (pneumonia). Prognosis: most patients die within a few weeks or months. A few remain locked-in for over a year before gradually recovering [5].

## Conclusion

We have presented here a case of locked-in syndrome in a Nigerian male with multiple sclerosis. The patient lies motionless and mute, yet conscious. The patient with the complete syndrome signals that he or she is conscious by opening and blinking the eyes and moving them in the vertical plane. Many clinicians have difficulty recognizing the syndrome while evaluating the “unconscious” patients, because they fail to routinely assess vertical eye movement and the patient’s ability to open and blink the eyes. Diagnosis is often made after patient’s family members or nursing staff call the attention of the doctor to the fact that the motionless and mute patient is in fact conscious and aware of his or her surroundings. The fact that our patient’s LIS was probably caused by a demyelinating disease (multiple sclerosis), a condition that is rare among blacks and in the African Continent made this case very interesting.

We do hope that our case report and the detailed literature review will further enhance easy recognition and management of the syndrome by all clinicians, especially those in Africa.
